# Association between ovarian sensitivity index and IVF-ET outcomes in women of advanced maternal age

**DOI:** 10.3389/fendo.2026.1889370

**Published:** 2026-07-24

**Authors:** Junjie Zhong, Lin Wang, Yudi Luo, Bowen Luo, Xiaofei Liu, Bojin Qin, Chun Yang, Juguang Zhang, Su’e Wei, Shuang Feng, Lingling Zhu, Zengyu Yang, Xiang Li

**Affiliations:** 1 Reproductive Medicine Center, Yulin Maternal and Child Health Hospital, Yulin, China; 2 Department of Clinical Laboratory, Yulin Maternal and Child Health Hospital, Yulin, China

**Keywords:** advanced-age women, clinical pregnancy, IVF-ET, ovarian responsiveness, ovarian sensitivity index

## Abstract

**Objective:**

To investigate the association between the ovarian sensitivity index (OSI) and ovarian responsiveness, embryo quality, and pregnancy outcomes in women of advanced reproductive age (≥35 years) undergoing IVF-ET.

**Methods:**

This retrospective cohort study included 1,055 fresh embryo transfer cycles performed at the Reproductive Medicine Center of Yulin Maternal and Child Health Hospital between January 1, 2021, and December 31, 2023. Patients were stratified by OSI tertiles: low (≤2.174, n=353), middle (2.174– 4.103, n=351), and high (>4.103, n=351). Multivariable logistic regression was used to adjust for confounders and assess the independent association between OSI and clinical pregnancy.

**Results:**

No significant differences were observed in infertility duration, BMI, LH, E2, T, P, or endometrial thickness on HCG day (all P>0.05). The high OSI group was younger with lower FSH and higher AMH/AFC (P<0.05), required less Gn and shorter stimulation, and yielded more oocytes, 2PN embryos, available embryos, and high-quality Day 3 embryos (P<0.05). Implantation and clinical pregnancy rates were higher in the high OSI group (P<0.05), but no differences were seen in live birth, multiple pregnancy, or early miscarriage rates (all P>0.05). Of note, the live birth rate — the most clinically meaningful endpoint in IVF — did not differ significantly across OSI tertiles (P = 0.412), indicating that the higher clinical pregnancy rates in the high OSI group did not translate into a higher live birth rate. After adjustment for age, FSH, AMH, and AFC, OSI tertiles were not independently associated with clinical pregnancy (overall P = 0.357). Age (OR = 0.880, 95% CI: 0.833–0.930, P<0.001) and AMH (OR = 1.114, 95% CI: 1.002–1.238, P = 0.046) remained independent predictors.

**Conclusion:**

In univariate analysis, higher OSI was associated with ovarian responsiveness, embryo quality, and clinical pregnancy rates; however, it is not independently associated with clinical pregnancy after adjustment for confounding factors, particularly age and AMH. OSI may serve as a composite indicator reflecting ovarian responsiveness and embryo quality, but should be interpreted in conjunction with age, AMH, and other relevant clinical factors.

## 1 Introduction

With the advancement of assisted reproductive technology (ART), fertility challenges in women of advanced reproductive age (≥35 years) have attracted increasing attention. Advancing maternal age is associated with a progressive decline in ovarian reserve and oocyte quality, which consequently leads to poor ovarian response, compromised embryo quality, and reduced clinical pregnancy rates in *in vitro* fertilization–embryo transfer (IVF-ET) cycles (1–3).

The ovarian sensitivity index (OSI) is an important indicator of ovarian responsiveness to exogenous gonadotropin stimulation in IVF-ET treatment, particularly in advanced-age women (4, 5). OSI is commonly defined as the ratio of the number of retrieved oocytes to the total gonadotropin dose and is calculated as follows: OSI = (number of retrieved oocytes/total gonadotropin dose) × 1000 (6). This index reflects the sensitivity of the ovaries to gonadotropin stimulation, with higher values indicating greater responsiveness to a given dose.

In advanced-age women, the clinical relevance of OSI is particularly pronounced, as this population frequently exhibits diminished ovarian reserve and reduced ovarian responsiveness (3). Ovarian aging is characterized by increased oxidative stress and impaired antioxidant defense mechanisms, which may influence OSI (1). Furthermore, age-related declines in oocyte quality (7), increased rates of embryonic aneuploidy, and altered endometrial receptivity may interact with OSI to collectively affect IVF-ET outcomes.

Therefore, the identification of reliable indicators capable of comprehensively evaluating ovarian responsiveness and guiding clinical decision-making is essential for facilitating individualized treatment strategies, optimizing resource allocation, and improving reproductive outcomes in advanced-age women.

## 2 Materials and methods

### 2.1 Study population and grouping

A total of 1,055 IVF-ET cycles performed between January 1, 2021, and December 31, 2023, were retrospectively included in this study.

The inclusion criteria were as follows (1): age ≥ 35 years (2); use of either a GnRH agonist or antagonist protocol (3); fertilization achieved via conventional IVF or intracytoplasmic sperm injection (ICSI); and (4) fresh embryo transfer cycles.

The exclusion criteria were as follows (1): irregular menstrual cycles or endocrine disorders (e.g., polycystic ovary syndrome) (2); conditions that may impair embryo implantation, including endometriosis, intrauterine adhesions, or endometrial tuberculosis (3); severe systemic diseases (4); current malignancy or a history of chemotherapy or radiotherapy (5); severe male factor infertility; and (6) known or suspected chromosomal abnormalities in either partner.

Patients were stratified into three groups according to OSI tertiles: low, middle, and high OSI groups (8). Specifically, the low OSI group was defined as OSI ≤ 2.174 (below the 33rd percentile, n = 353), the middle OSI group as 2.174 < OSI ≤ 4.103 (33rd–67th percentile, n = 351), and the high OSI group as OSI > 4.103 (above the 67th percentile, n = 351).

### 2.2 Ovulation induction methods

GnRH agonist protocol: A long-acting GnRH agonist (leuprorelin, Livzon Pharmaceutical, China) was administered at a dose of 3.75 mg on days 2–3 of menstruation. After 28–35 days, recombinant follicle-stimulating hormone (rFSH) was initiated if pituitary downregulation criteria were met [serum luteinizing hormone (LH) < 5 mIU/L, estradiol (E2) < 50 pg/mL, and follicle diameter < 10 mm]. rFSH preparations included Gonal-f (Merck Serono, Germany) and Jin Saiheng (Changchun JinSai Pharmaceutical, China).

GnRH antagonist protocol: Recombinant FSH was initiated on days 2–3 of menstruation. A GnRH antagonist (0.25 mg/day) was introduced when any of the following criteria were met: follicle diameter ≥ 14 mm, E2 ≥ 300 pg/mL, or LH ≥ 10 IU/mL, and was continued until the trigger day. Ovulation was triggered with human chorionic gonadotropin (HCG) when one dominant follicle reached a mean diameter of 18 mm or when two dominant follicles reached 17 mm. Oocyte retrieval was performed 34–36 hours later under transvaginal ultrasound guidance. Fertilization was achieved via either conventional IVF or ICSI, depending on sperm quality.

### 2.3 Embryo assessment and transfer

Embryos were evaluated according to the Istanbul consensus criteria (9). Embryo transfer was performed under ultrasound guidance on day 3 after oocyte retrieval. One or two top-quality Day 3 embryos were transferred. Surplus viable embryos were either cryopreserved immediately or cultured to the blastocyst stage prior to cryopreservation.

Luteal phase support was initiated on the day of oocyte retrieval using vaginal progesterone administered three times daily. Biochemical pregnancy was determined by serum HCG measurement 14 days after embryo transfer, and clinical pregnancy was confirmed by transvaginal ultrasound 34 days after transfer. Live birth was defined as the delivery of a live neonate.

### 2.4 Outcome measures

Baseline characteristics included age, body mass index (BMI), duration of infertility, baseline hormone levels [follicle-stimulating hormone (FSH), LH, E2, testosterone (T), progesterone (P), anti-Müllerian hormone (AMH)], antral follicle count (AFC), and endometrial thickness on the day of HCG administration. Ovarian response parameters included duration of gonadotropin (Gn) stimulation, total Gn dose, and number of retrieved oocytes. Embryological parameters included the number of two-pronuclear (2PN) embryos, available Day 3 embryos, and high-quality Day 3 embryos.

Pregnancy outcomes included clinical pregnancy rate (number of clinical pregnancy cycles/number of transfer cycles × 100%), implantation rate (number of implanted embryos/number of transferred embryos × 100%), multiple pregnancy rate (number of multiple pregnancy cycles/number of clinical pregnancy cycles × 100%), early miscarriage rate (number of early miscarriage cycles/number of clinical pregnancy cycles × 100%), and live birth rate (number of live birth cycles/number of transfer cycles × 100%).

### 2.5 Statistical analysis

Statistical analyses were performed using SPSS version 25.0 (IBM Corp., Armonk, NY, USA).

Categorical variables were expressed as percentages (%) and compared using the chi-square (χ²) test. Continuous variables with normal distribution were presented as mean ± standard deviation (SD) and compared using one-way analysis of variance (ANOVA).

To control for baseline differences among OSI groups, including age, baseline FSH, AMH, and AFC, binary logistic regression analysis was conducted to evaluate the independent association between OSI and clinical pregnancy. Clinical pregnancy (1 = yes, 0 = no) was set as the dependent variable. Independent variables included age (continuous), baseline FSH (continuous), baseline AMH (continuous), AFC (continuous), and OSI tertiles (with the low OSI group as the reference). All variables were entered into the model using the Enter method.

Model goodness-of-fit was assessed using the Hosmer–Lemeshow test. Results were reported as adjusted odds ratios (ORs) with 95% confidence intervals (CIs). All statistical tests were two-sided, and P < 0.05 was considered statistically significant.

To assess the robustness of our findings, we performed several supplementary analyses (1): OSI was entered as a continuous variable in the multivariable logistic regression model (2); trend analysis across OSI tertiles was tested (unadjusted and adjusted) (3); subgroup analysis was performed according to AMH level (<1.5 ng/mL vs. ≥1.5 ng/mL). All supplementary models adjusted for age, baseline FSH, AMH, AFC, fertilization type, double embryo transfer, and COH protocol (three categories: luteal−phase long, follicular−phase long, antagonist), and included OSI×age and OSI×AMH interaction terms. Results are presented in **Supplementary Tables 1**–**3**.Covariates (age, baseline FSH, AMH, AFC) were selected based on biological plausibility and prior literature. Multicollinearity among continuous predictors was assessed using variance inflation factors (VIF); all VIF values were below 2, indicating no significant collinearity. Interaction terms (OSI × age and OSI × AMH) were tested in the main model and were not significant (P > 0.05 for both).

## 3 Results

### 3.1 Baseline characteristics across OSI groups

No statistically significant differences were observed among the three groups in infertility duration, body mass index (BMI), baseline luteinizing hormone (LH), estradiol (E2), testosterone (T), progesterone (P), or endometrial thickness on the day of human chorionic gonadotropin (HCG) administration (all P > 0.05). Compared with the low OSI group, patients in the middle and high OSI groups were younger and exhibited lower baseline follicle-stimulating hormone (FSH) levels, higher anti-Müllerian hormone (AMH) levels, and higher antral follicle counts (AFC) (all P < 0.05). Detailed results are presented in **Table 1**.

**Table 1:** Baseline characteristics of the three groups (mean ± SD).

Characteristic	Low OSI group (n=353) (≤2.174)	Middle OSI group (n=351) (2.175–4.103)	High OSI group (n=351) (≥4.104)	F/χ2	P value
Age (years)	38.29	37.66	37.21	17.872	<0.001
Infertility duration (years)	5.13	5.06	5.3	0.182	0.834
BMI (kg/m²)	22.75	22.62	22.47	0.839	0.432
Basal FSH (mIU/mL)	7.18	6.37	5.61	36.921	<0.001
Basal LH (mIU/mL)	3.04	3.27	3.46	2.95	0.053
Basal E2 (pg/mL)	66.02	79.6	63.54	0.533	0.587
Basal T (ng/mL)	0.34	0.35	0.42	0.247	0.781
Basal P (ng/mL)	0.72	0.64	0.77	0.227	0.797
Basal AMH (ng/mL)	1.63	2.25	3.35	145.59	<0.001
AFC (n)	9.52	11.81	15.78	141.982	<0.001
Endometrial thickness on hCG day (mm)	10.46	10.61	10.91	2.98	0.051

### 3.2 Ovarian response and embryo quality across OSI groups

Significant differences were observed among the three groups in preovulatory follicle count (PFC), number of retrieved oocytes, number of two-pronuclear (2PN) embryos, number of available embryos, and number of high-quality Day 3 embryos (all P < 0.05). These parameters were lowest in the low OSI group, intermediate in the middle OSI group, and highest in the high OSI group.

In contrast, the total gonadotropin (Gn) dose and duration of stimulation were significantly greater in the low OSI group than in the middle and high OSI groups (all P < 0.05). Detailed results are shown in **Table 2**.

**Table 2:** Ovarian response and embryo quality among the three groups (mean ± SD).

Characteristic	Low OSI group (n=353) (≤2.174)	Middle OSI group (n=351) (2.175–4.103)	High OSI group (n=351) (≥4.104)	F/χ2	P value
Total Gn dose (IU)	3146.84	2801.82	2232.19	138.126	<0.001
Duration of stimulation (days)	11.07	10.67	10.35	9.842	<0.001
No. of pre-ovulatory follicles (PFC)	4.98	7.6	10.26	222.066	<0.001
No. of oocytes retrieved	4.44	8.48	13.55	1060.14	<0.001
No. of 2PN zygotes	3	5.5	8.35	368.081	<0.001
No. of usable embryos	2.94	5	7.97	349.532	<0.001
No. of top-quality D3 embryos	1.73	2.72	4.41	169.206	<0.001

### 3.3 Pregnancy outcomes following fresh embryo transfer

No significant differences were observed among the three groups in multiple pregnancy rate, early miscarriage rate, or live birth rate (all P > 0.05). However, the implantation rate and clinical pregnancy rate were significantly higher in the high OSI group than in the middle and low OSI groups (all P < 0.05).

The mean number of embryos transferred was highest in the middle OSI group, followed by the high OSI group, and lowest in the low OSI group, with statistically significant differences among groups (P < 0.05). Importantly, despite the stepwise increase in clinical pregnancy rates from the low to the high OSI group (38.8%, 47.3%, and 51.3%), the live birth rate did not differ significantly across the three groups (P = 0.412). This dissociation between clinical pregnancy and live birth suggests that the higher clinical pregnancy rate in the high OSI group does not translate into a higher live birth rate. Detailed results are presented in **Table 3**.

**Table 3:** Pregnancy outcomes among the three groups.

Outcome	Low OSI group (n=353) (≤2.174)	Middle OSI group (n=351) (2.175–4.103)	High OSI group (n=351) (≥4.104)	F/χ2	P value
Implantation rate (%)	26.89 (164/610)	33.33 (213/639)	36.08 (223/618)	8.277	0.016
Clinical pregnancy rate (%)	38.81 (137/353)	47.29 (166/351)	51.28 (180/351)	5.803	0.003
Multiple pregnancy rate (%)	13.87 (19/137)	16.27 (27/166)	13.89 (25/180)	0.227	0.797
Early miscarriage rate (%)	16.42 (22/137)	19.28 (32/166)	20.00 (36/180)	0.609	0.544
Live birth rate (%)	30.31 (107/353)	34.76 (122/351)	36.75 (129/351)	0.89	0.412
Mean number of embryos transferred	1.73	1.82	1.76	4.388	0.013

### 3.4 Multivariable logistic regression analysis

To adjust for baseline differences among OSI groups, including age, baseline FSH, AMH, and AFC, a binary logistic regression analysis was performed to evaluate the independent association between OSI and clinical pregnancy. Clinical pregnancy was included as the dependent variable, while age, baseline FSH, baseline AMH, AFC, and OSI tertiles (with the low OSI group as the reference) were entered simultaneously into the model. The overall effect of OSI tertiles was not statistically significant (overall test P = 0.357). Compared with the low OSI group, neither the middle OSI group (OR = 0.815, 95% CI: 0.571–1.162, P = 0.259) nor the high OSI group (OR = 1.011, 95% CI: 0.733–1.395, P = 0.946) showed a significant association with clinical pregnancy (**Table 4**). 

**Table 4:** Multivariate logistic regression analysis of factors affecting clinical pregnancy in advanced−age women undergoing IVF.

Variable	B	OR	95% CI	P value
Age (years)	−0.128	0.88	0.833–0.930	<0.001
Basal FSH (mIU/mL)	0.011	1.011	0.959–1.066	0.68
Basal AMH (ng/mL)	0.108	1.114	1.002–1.238	0.046
AFC (n)	0.002	1.002	0.973–1.031	0.896
OSI tertiles†				0.357
Middle vs. Low OSI	−0.205	0.815	0.571–1.162	0.259
High vs. Low OSI	0.011	1.011	0.733–1.395	0.946
Constant	4.354	77.789	—	<0.001

† OSI tertiles were entered as a categorical variable; the low OSI group served as the reference.

Baseline FSH (P = 0.680) and AFC (P = 0.896) were also not independently associated with clinical pregnancy. The Hosmer–Lemeshow goodness-of-fit test yielded χ² = 9.584 (df = 8, P = 0.295), indicating good model calibration, with no significant difference between predicted and observed outcomes.

### 3.5 Supplementary analyses

When OSI was analyzed as a continuous variable in the fully adjusted model, it was not independently associated with clinical pregnancy (OR = 0.713, 95% CI: 0.275–1.849, P = 0.486) (**Supplementary Table 1**). Trend analysis showed a significant unadjusted linear trend across OSI tertiles (P = 0.001), which was completely attenuated after multivariable adjustment (P = 0.825) (**Supplementary Table 2**). Subgroup analysis by AMH level revealed that OSI tertiles were not significantly associated with clinical pregnancy in either the low AMH (<1.5 ng/mL) or normal AMH (≥1.5 ng/mL) subgroup (overall P = 0.677 and 0.416, respectively) (**Supplementary Table 3**).

## 4 Discussion

This study retrospectively analyzed 1,055 IVF-ET cycles in advanced-age women and stratified patients according to OSI tertiles to systematically investigate the associations between OSI and ovarian response, embryo quality, and pregnancy outcomes. The findings demonstrated that patients in the high OSI group were younger and exhibited lower baseline follicle-stimulating hormone (FSH) levels, higher anti-Müllerian hormone (AMH) levels, and greater antral follicle counts (AFC), indicating superior ovarian reserve. During ovarian stimulation, the high OSI group showed enhanced ovarian responsiveness, as reflected by higher numbers of retrieved oocytes, two-pronuclear (2PN) embryos, available embryos, and high-quality embryos, while requiring significantly lower gonadotropin (Gn) doses and shorter stimulation duration.

Univariate analysis indicated that the high OSI group had significantly higher implantation and clinical pregnancy rates than the low OSI group; however, no significant differences were observed among the three groups in live birth rate, multiple pregnancy rate, or early miscarriage rate. This absence of a significant difference in live birth rate — the most clinically meaningful endpoint in ART — is a key finding of our study. It critically limits the clinical utility of OSI as a prognostic tool for predicting live birth, despite its association with intermediate outcomes such as clinical pregnancy. After adjustment for age, baseline FSH, AMH, and AFC in multivariable logistic regression, the independent association between OSI and clinical pregnancy was no longer significant (overall test P = 0.357), whereas age and AMH remained significant predictors. These findings suggest that the association between OSI and clinical pregnancy observed in univariate analysis is largely confounded by age and ovarian reserve markers, and that OSI is not independently associated with clinical pregnancy beyond these conventional indicators. This has important implications for the clinical interpretation of OSI.

Previous studies have reported associations between OSI and pregnancy outcomes in advanced-age women (5, 10, 11). For instance, a retrospective cohort study of women aged ≥39 years demonstrated that OSI was associated with cumulative pregnancy rate and live birth rate (5), while another study reported significant associations between OSI and both clinical pregnancy and live birth rates (11). However, many of these studies did not adequately adjust for key confounders such as age and AMH or were limited by relatively small sample sizes. In the present study, multivariable adjustment in a relatively large cohort attenuated the association between OSI and clinical pregnancy, indicating that the clinical value of OSI may primarily lie in its role as a composite indicator of ovarian reserve and responsiveness rather than as an independent predictor.

When assessing ovarian responsiveness in advanced-age women, OSI offers certain advantages over other indices, such as the follicular output rate (FORT) and the follicle–oocyte index (FOI) (12); however, the independent predictive value of these indices remains uncertain. A modified ovarian sensitivity index (MOSI), incorporating initial follicular measurements and starting FSH dose, has been proposed as a predictor of high-quality embryo yield (13), which may be particularly relevant in this population. Future studies should explore the incremental predictive value of MOSI after accounting for confounding factors such as age and AMH.

The primary clinical relevance of OSI lies in its potential to guide individualized ovarian stimulation strategies. A low OSI reflects reduced ovarian sensitivity, and the present findings indicate that such patients require higher Gn doses and longer stimulation durations while achieving poorer outcomes. Accordingly, protocol optimization should be considered, including increasing the initial Gn dose or adopting alternative approaches such as luteal-phase stimulation or progestin-primed ovarian stimulation (PPOS) (1). Notably, ovarian reserve and chronological age are not always concordant. Younger patients with diminished ovarian reserve may exhibit relatively higher ovarian sensitivity, whereas older patients with relatively preserved ovarian reserve may demonstrate reduced sensitivity. This heterogeneity underscores the need for individualized clinical management.

Combining OSI with established ovarian reserve markers such as AMH may provide a more comprehensive assessment of ovarian function and improve prediction of ovarian response. However, OSI should not be used as an independent predictor of pregnancy outcomes separate from age and AMH. In specific populations, the utility of OSI may be more pronounced. For example, in patients with polycystic ovary syndrome (PCOS), OSI has been shown to be negatively correlated with insulin resistance (HOMA-IR) (10, 14, 15), suggesting that metabolic factors play a critical role in ovarian responsiveness. Although PCOS is less common in advanced-age women, age-related metabolic alterations may still influence OSI. A large retrospective cohort study involving 2,055 patients with PCOS indicated that insulin resistance may affect IVF outcomes through its impact on OSI (15).

Furthermore, in patients classified as having poor prognosis according to the POSEIDON criteria, OSI may help identify subgroups more likely to benefit from individualized interventions, thereby facilitating more precise treatment strategies (1). Ovarian stimulation protocols may also influence OSI in advanced-age women. Luteal-phase ovarian stimulation has been investigated as an alternative following failure of long or ultra-long protocols and may offer additional options (16). Additionally, the application of GnRH antagonist protocols in patients with normal ovarian response also involves consideration of OSI (17, 18).

An important consideration is the potential confounding effect of embryo transfer strategy. In our cohort, the mean number of embryos transferred differed significantly across OSI groups (P = 0.013), with the middle OSI group receiving the highest average number. To address this, we included the number of embryos transferred (single vs. double) as a covariate in the multivariable model. Double embryo transfer was strongly associated with clinical pregnancy (OR = 1.991, 95% CI: 1.461–2.713, P < 0.001; **Supplementary Table 1**), confirming that transfer strategy is a major determinant of pregnancy success. Nevertheless, after adjusting for this factor, OSI remained non−significant. This indicates that the null association between OSI and clinical pregnancy is not explained by differences in transfer strategy. Importantly, the more aggressive transfer strategy in the middle OSI group did not translate into a higher live birth rate, further supporting that clinical decision−making regarding transfer number does not confound our primary conclusion. Future strategies should incorporate more refined embryo selection methods, such as time-lapse imaging or blastocyst culture, to optimize outcomes.

The lack of association between OSI and live birth deserves further emphasis. Live birth is universally recognized as the ultimate success metric in IVF. In our cohort, even though the high OSI group achieved significantly more oocytes, better embryos, and higher clinical pregnancy rates, this advantage was completely lost by the time of live birth. This observation suggests that factors beyond the reach of OSI — such as endometrial receptivity, embryo aneuploidy, or late pregnancy complications — may play decisive roles in determining live birth. Consequently, OSI should not be used to counsel patients regarding their chances of taking home a baby; its role remains limited to describing ovarian sensitivity and guiding stimulation strategies.

This study has several limitations. First, as a single-center retrospective study, it is subject to potential selection bias despite the relatively large sample size. Second, the absence of key data, such as embryo aneuploidy rates (e.g., PGT-A), precludes evaluation of the relationship between OSI and embryonic genetic quality. Third, although multivariable regression was used to adjust for known confounders, residual confounding from unmeasured factors (e.g., lifestyle and genetic influences) cannot be excluded. Fourth, although we adjusted for the number of embryos transferred (single vs. double), we could not account for the exact reasons behind transfer decisions (e.g., embryo quality scores, patient preference), which may introduce residual confounding. However, the strong association between double embryo transfer and clinical pregnancy (OR ≈ 2.0) suggests that our adjustment captured the major effect of transfer strategy. Future research should focus on developing more precise indices, such as MOSI or the follicular sensitivity index (FSI), validating the role of OSI in individualized treatment through large-scale, multicenter prospective studies, and exploring its direct relationship with oocyte quality and embryonic developmental potential.

## 5 Conclusion

The ovarian sensitivity index (OSI) is a valuable composite indicator for assessing ovarian responsiveness and embryo quality in advanced-age women. Although univariate analysis suggests that higher OSI is associated with improved implantation and clinical pregnancy rates, this association is not independent after adjustment for confounding factors such as age and AMH. Moreover, OSI was not associated with live birth rate, the most clinically meaningful endpoint. Age and baseline AMH remain the primary independent predictors of clinical pregnancy in this population.

Therefore, OSI should not be applied in isolation but rather interpreted in conjunction with patient age, ovarian reserve markers, and other clinical parameters, and incorporated into individualized ovarian stimulation and embryo transfer strategies. Despite its integrative value, OSI is influenced by confounding factors and should not be considered a standalone predictive tool. Future studies should aim to refine this index and validate its clinical utility through prospective investigations.

## Data Availability

The data can be obtained from the author. Requests to access the datasets should be directed to Junjie Zhong, zhongjunjie074@163.com.
