# Supplementary material for: Association between ovarian sensitivity index and IVF-ET outcomes in women of advanced maternal age
Source: Front Endocrinol (Lausanne). 2026 Jul 24;17:1889370. doi: 10.3389/fendo.2026.1889370 (PMC13447226; doi:10.3389/fendo.2026.1889370)
Supplement: Supplementary file 1 [file Table1.docx]

Supplementary Tables S1–S3 for: Association Between Ovarian Sensitivity Index and IVF-ET Outcomes in Women of Advanced Reproductive Age

| Supplementary Table S1. Multivariable logistic regression analysis for clinical pregnancy with OSI as a continuous variable | | | |
| --- | --- | --- | --- |
| Variable | B | OR (95% CI) | P value |
| Double embryo transfer (ref: single) | 0.689 | 1.991 (1.461–2.713) | <0.001 |
| OSI value (per 1-unit increase) | -0.338 | 0.713 (0.275–1.849) | 0.486 |
| Age (years) | -0.167 | 0.846 (0.762–0.939) | 0.002 |
| Baseline FSH (mIU/mL) | 0.016 | 1.016 (0.964–1.071) | 0.547 |
| AMH (ng/mL) | 0.146 | 1.157 (0.982–1.364) | 0.081 |
| AFC (count) | -0.005 | 0.995 (0.965–1.027) | 0.769 |
| OSI × age interaction | 0.01 | 1.010 (0.985–1.036) | 0.422 |
| OSI × AMH interaction | -0.003 | 0.997 (0.972–1.023) | 0.829 |
| Fertilization type (ref: ICSI) |  |  |  |
| IVF vs. ICSI | 0.062 | 1.064 (0.797–1.422) | 0.673 |
| COH protocol (ref: antagonist) |  |  | Overall P = 0.078 |
| Luteal‑phase long vs. antagonist | 0.337 | 1.401 (0.995–1.973) | 0.054 |
| Follicular‑phase long vs. antagonist | 0.071 | 1.074 (0.745–1.548) | 0.703 |
| Constant | 4.839 | 126.284 | 0.019 |
| *Notes: Dependent variable: clinical pregnancy (1=yes, 0=no). Hosmer–Lemeshow test P = 0.205, indicating good model fit. COH protocol coding: 1 = luteal‑phase long, 2 = follicular‑phase long, 3 = antagonist.* | | | |

| Supplementary Table S2. Trend analysis of clinical pregnancy across OSI tertiles | | | | |
| --- | --- | --- | --- | --- |
| OSI tertile | n | Clinical pregnancy rate (%) | Unadjusted OR (95% CI) | Adjusted OR (95% CI)* |
| Low (≤2.174) | 353 | 38.8 (137/353) | 1.00 (ref) | 1.00 (ref) |
| Middle (2.174–4.103) | 351 | 47.3 (166/351) | 1.42 (1.05–1.92) | 0.93 (0.47–1.81) |
| High (>4.103) | 351 | 51.3 (180/351) | 1.66 (1.23–2.24) | 1.02 (0.64–1.64) |
| P for trend |  |  | 0.001 | 0.825 |
| Adjusted for age, baseline FSH, AMH, AFC, fertilization type, double embryo transfer, COH protocol, and OSI×age and OSI×AMH interactions. Unadjusted P for trend was obtained from the linear‑by‑linear association test (Pearson R). Adjusted P for trend was derived from the logistic regression model with OSI tertiles entered as a continuous ordinal variable (1,2,3). | | | | |

| Supplementary Table S3. Subgroup analysis by AMH level: Association between OSI tertiles and clinical pregnancy | | | | |
| --- | --- | --- | --- | --- |
| Subgroup | n | Overall P for OSI tertiles | OR (95% CI) for middle vs. low OSI | OR (95% CI) for high vs. low OSI |
| Low AMH (<1.5 ng/mL) | 317 | 0.677 | 1.000 (0.368–2.715) | 1.259 (0.450–3.523) |
| Normal AMH (≥1.5 ng/mL) | 738 | 0.416 | 0.757 (0.497–1.153) | 0.866 (0.611–1.228) |
| *Notes: Both subgroups were adjusted for age, baseline FSH, AFC, fertilization type (IVF/ICSI), double embryo transfer, and COH protocol (three categories, with antagonist as reference). The overall effect of COH protocol was not significant in either subgroup (P > 0.05).* | | | | |
